# Supplementary material for: Comparison of nutritional supplements in improving glycolipid metabolism and endocrine function in polycystic ovary syndrome: a systematic review and network meta-analysis
Source: PeerJ. 2023 Nov 13;11:e16410. doi: 10.7717/peerj.16410 (PMC10652859; doi:10.7717/peerj.16410)
Supplement: Supplemental Information 2 [file peerj-11-16410-s002.docx]

# Supplementary material

**TableS1 search strategy**

| No. | Query | Results |
| --- | --- | --- |
| **Pubmed** | | |
| 1 | "Polycystic Ovary Syndrome"[Mesh] | 17552 |
| 2 | 'cystic ovary'[Title/Abstract] OR 'micropolycystic ovary'[Title/Abstract] OR 'multiple follicle cyst'[Title/Abstract] OR 'ovary polycystic'[Title/Abstract] OR 'stein cohen leventhal syndrome'[Title/Abstract] OR 'stein leventhal'[Title/Abstract] OR 'syndrome stein leventhal'[Title/Abstract] OR ' Polycystic Ovar*'[Title/Abstract] OR ' Sclerocystic Ovar*'[Title/Abstract] OR 'PCOS'[Title/Abstract] | 15694 |
| 3 | ('cystic ovary'[Title/Abstract] OR 'micropolycystic ovary'[Title/Abstract] OR 'multiple follicle cyst'[Title/Abstract] OR 'ovary polycystic'[Title/Abstract] OR 'stein cohen leventhal syndrome'[Title/Abstract] OR 'stein leventhal'[Title/Abstract] OR 'syndrome stein leventhal'[Title/Abstract] OR ' Polycystic Ovar*'[Title/Abstract] OR ' Sclerocystic Ovar*'[Title/Abstract] OR 'PCOS'[Title/Abstract]) OR ("Polycystic Ovary Syndrome"[Mesh]) | 21090 |
| 4 | ‘inositol’[Title/Abstract] OR ‘Coenzyme Q10’[Title/Abstract] OR ‘vitamin E’[Title/Abstract] OR ‘tocopherol’[Title/Abstract] OR ‘tocotrienol’[Title/Abstract] OR ‘omega-3’[Title/Abstract] OR ‘n-3 fatty acid’[Title/Abstract] OR ‘W-3’[Title/Abstract] OR ‘EPA’[Title/Abstract] OR ‘DHA’[Title/Abstract] OR ‘ALA’[Title/Abstract] OR ‘fish oil’[Title/Abstract] OR ‘alpha-Linolenic Acid’[Title/Abstract] OR ‘Docosahexaenoic Acids’[Title/Abstract] OR ‘Eicosapentaenoic Acid’[Title/Abstract] OR ‘vitamin D’[Title/Abstract] OR ‘chromium’[Title/Abstract] OR ‘Selenium’[Title/Abstract] OR ‘carnitine’[Title/Abstract] OR ‘levocarnitine’[Title/Abstract] OR ‘vitamin BT’[Title/Abstract] OR ‘L carnitine’[Title/Abstract] OR ‘bicarnesine’[Title/Abstract] OR ‘Probiotics’[Title/Abstract] | 359587 |
| 5 | ‘RCT’ OR ‘random*’ OR ‘randomized controlled trial’ | 1196337 |
| 6 | ((('cystic ovary'[Title/Abstract] OR 'micropolycystic ovary'[Title/Abstract] OR 'multiple follicle cyst'[Title/Abstract] OR 'ovary polycystic'[Title/Abstract] OR 'stein cohen leventhal syndrome'[Title/Abstract] OR 'stein leventhal'[Title/Abstract] OR 'syndrome stein leventhal'[Title/Abstract] OR ' Polycystic Ovar*'[Title/Abstract] OR ' Sclerocystic Ovar*'[Title/Abstract] OR 'PCOS'[Title/Abstract]) OR ("Polycystic Ovary Syndrome"[Mesh])) AND (‘inositol’[Title/Abstract] OR ‘Coenzyme Q10’[Title/Abstract] OR ‘vitamin E’[Title/Abstract] OR ‘tocopherol’[Title/Abstract] OR ‘tocotrienol’[Title/Abstract] OR ‘omega-3’[Title/Abstract] OR ‘n-3 fatty acid’[Title/Abstract] OR ‘W-3’[Title/Abstract] OR ‘EPA’[Title/Abstract] OR ‘DHA’[Title/Abstract] OR ‘ALA’[Title/Abstract] OR ‘fish oil’[Title/Abstract] OR ‘alpha-Linolenic Acid’[Title/Abstract] OR ‘Docosahexaenoic Acids’[Title/Abstract] OR ‘Eicosapentaenoic Acid’[Title/Abstract] OR ‘vitamin D’[Title/Abstract] OR ‘chromium’[Title/Abstract] OR ‘Selenium’[Title/Abstract] OR ‘carnitine’[Title/Abstract] OR ‘levocarnitine’[Title/Abstract] OR ‘vitamin BT’[Title/Abstract] OR ‘L carnitine’[Title/Abstract] OR ‘bicarnesine’[Title/Abstract] OR ‘Probiotics’[Title/Abstract])) AND (‘RCT’ OR ‘random*’ OR ‘randomized controlled trial’) | 198 |
| **Embase** | | |
| 1 | 'ovary polycystic disease'/exp OR 'ovary polycystic disease' | 35678 |
| 2 | 'cystic ovary':ab,ti OR 'micropolycystic ovary':ab,ti OR 'multiple follicle cyst':ab,ti OR 'ovary polycystic':ab,ti OR 'stein cohen leventhal syndrome':ab,ti OR 'stein leventhal':ab,ti OR 'syndrome stein leventhal':ab,ti OR 'polycystic ovar*':ab,ti OR 'sclerocystic ovar*':ab,ti OR 'pcos':ab,ti | 32708 |
| 3 | #1 OR #2 | 40599 |
| 4 | 'inositol':ab,ti OR 'coenzyme q10':ab,ti OR 'vitamin e':ab,ti OR 'tocopherol':ab,ti OR 'tocotrienol':ab,ti OR 'omega-3':ab,ti OR 'n-3 fatty acid':ab,ti OR 'w-3':ab,ti OR 'epa':ab,ti OR 'dha':ab,ti OR 'ala':ab,ti OR 'fish oil':ab,ti OR 'alpha-linolenic acid':ab,ti OR 'docosahexaenoic acids':ab,ti OR 'eicosapentaenoic acid':ab,ti OR 'vitamin d':ab,ti OR 'chromium':ab,ti OR 'selenium':ab,ti OR 'carnitine':ab,ti OR 'levocarnitine':ab,ti OR 'vitamin bt':ab,ti OR 'l carnitine':ab,ti OR 'bicarnesine':ab,ti OR 'probiotic':ab,ti | 449267 |
| 5 | 'rct' OR 'random*' OR 'randomized controlled trial' | 2173564 |
| 6 | #3 AND #4 AND #5 | 479 |
| **Web of science** | | |
| 1 | (TI=('cystic ovary' OR 'micropolycystic ovary' OR 'multiple follicle cyst' OR 'ovary polycystic' OR 'stein cohen leventhal syndrome' OR 'stein leventhal' OR 'syndrome stein leventhal' OR ' Polycystic Ovar*' OR ' Sclerocystic Ovar*' OR 'PCOS')) OR AB=('cystic ovary' OR 'micropolycystic ovary' OR 'multiple follicle cyst' OR 'ovary polycystic' OR 'stein cohen leventhal syndrome' OR 'stein leventhal' OR 'syndrome stein leventhal' OR ' Polycystic Ovar*' OR ' Sclerocystic Ovar*' OR 'PCOS') | 38939 |
| 2 | TI=('inositol' OR 'Coenzyme Q10' OR 'vitamin E' OR 'tocopherol' OR 'tocotrienol' OR 'omega-3' OR 'n-3 fatty acid' OR 'W-3' OR 'EPA' OR 'DHA' OR 'ALA' OR 'fish oil' OR 'alpha-Linolenic Acid' OR 'Docosahexaenoic Acids' OR 'Eicosapentaenoic Acid' OR 'vitamin D' OR 'chromium' OR 'Selenium' OR 'carnitine' OR 'levocarnitine' OR 'vitamin BT' OR 'L carnitine' OR 'bicarnesine' OR 'Probiotic') | 534394 |
| 3 | (TI=('RCT' OR 'random*' OR 'randomized controlled trial')) OR AB=('RCT' OR 'random*' OR 'randomized controlled trial') | 3031680 |
| 4 | #3 AND #2 AND #1 | 286 |
| **Cochrane** | | |
| 1 | MeSH descriptor: [Polycystic Ovary Syndrome] explode all trees | 1866 |
| 2 | ('cystic ovary' OR 'micropolycystic ovary' OR 'multiple follicle cyst' OR 'ovary polycystic' OR 'stein cohen leventhal syndrome' OR 'stein leventhal' OR 'syndrome stein leventhal' OR ' Polycystic Ovar*' OR ' Sclerocystic Ovar*' OR 'PCOS'):ti,ab,kw in Cochrane Reviews | 40 |
| 3 | #1 OR #2 in Cochrane Reviews | 40 |
| 4 | ('inositol' OR 'Coenzyme Q10' OR 'vitamin E' OR 'tocopherol' OR 'tocotrienol' OR 'omega-3' OR 'n-3 fatty acid' OR 'W-3' OR 'EPA' OR 'DHA' OR 'ALA' OR 'fish oil' OR 'alpha-Linolenic Acid' OR 'Docosahexaenoic Acids' OR 'Eicosapentaenoic Acid' OR 'vitamin D' OR 'chromium' OR 'Selenium' OR 'carnitine' OR 'levocarnitine' OR 'vitamin BT' OR 'L carnitine' OR 'bicarnesine' OR 'Probiotic'):ti,ab,kw in Cochrane Reviews | 467 |
| 5 | ('RCT' OR 'random*' OR 'randomized controlled trial') in Cochrane Reviews | 8842 |
| 6 | #3 AND #4 AND #5 in Cochrane Reviews | 2 |

**TableS2 The basic characteristics of the included literature**

| **Study** | **Country** | **Sample size** | | **Intervention** | | **Duration** | **Age** | | **BMI** | | **Outcome indicators** |
| --- | --- | --- | --- | --- | --- | --- | --- | --- | --- | --- | --- |
|  |  | **T** | **C** | **T** | **C** |  | **T** | **C** | **T** | **C** |  |
| Hamidreza et al.2017 | Iran | 30 | 30 | Carnitine 250mg/qd | placebo/qd | 12w | 27.1±5.2 | 27.2±5.1 | 29.0±3.5 | 28.0±4.4 | 1,2 |
| Mehrnush et al.2018 | Iran | 20 | 20 | Chromium 200µg/qd | placebo/qd | 8w | 33.3±2.7 | 33.8±1.9 | 27.7±2.5 | 27.0±3.9 | 1,2,13 |
| Mehri et al.2015 | Iran | 32 | 32 | Chromium 200μg/qd | placebo/qd | 8w | 24.9±5.0 | 24.4±4.4 | 25.8±5.1 | 25.4±4.0 | 1,2,3,4,5,6,7,8,9,10 |
| Mehri et al.2018 | Iran | 20 | 20 | Chromium 200μg/qd | placebo/qd | 8w | 30.3±4.6 | 32.3±3.0 | 27.4±3.4 | 26.6±5.1 | 1,2,3,4,5,6,7,8,9,10 |
| R. Scott et al.2005 | USA | 6 | 4 | Chromium 200μg/qd | placebo/qd | 16w | 18-39 | 18-39 | - | - | 3,4,7,8,11 |
| Sherif et al.2016 | Egypt | 44 | 41 | Chromium 1000μg/qd | placebo/qd | 24w | 24.7±3.7 | 24.6±4 | 30±3.3 | 29.7±3.1 | 2,3,4,11 |
| Maryam et al.2022 | Iran | 28 | 27 | Co Q10 100mg/qd | placebo/qd | 12w | 27.1±6.5 | 29.0±6.9 | 27.2±3.9 | 28.2±4.8 | 1,2,11,12,13 |
| Elham et al.2017 | Iran | 20 | 20 | Co Q10 100mg/qd | placebo/qd | 12w | 24.9±3.7 | 24.7±5.3 | 27.7±3.6 | 28.9±6.6 | 1,2 |
| Shahad et al.2022 | Iraq | 50 | 50 | Co Q10 200mg/qd | placebo/qd | 12w | 25-33 | 25-33 | 29.14±6.96 | 30.10±5.40 | 2,3,7,8,9,10 |
| Gabriella et al.2012 | Italy | 18 | 8 | Inositol 1200mg/qd | placebo/qd | 12w | 23.5±2.1 | 23.6±1.4 | 21.6±1.9 | 21.9±0.6 | 1,2,3,4,5 |
| Mehrdad et al.2018 | Iran | 27 | 27 | Omega-3 2×1000mg/qd | placebo/qd | 12w | 27.2±6.2 | 28.9±4.2 | 25.8±4.8 | 25.9±4.3 | 1,2,3,4,5,6,7,8,9,10,11,12,13 |
| Azadeh et al.2015 | Iran | 39 | 39 | Omega-3 3×1g/qd | placebo 3×1g/qd | 8w | 26.9±5.9 | 26.9±5.0 | 31.46±5.74 | 31.88±3.86 | 2 |
| Elahe et al.2012 | Iran | 30 | 31 | Omega-3 4×1000mg/qd | placebo/qd | 8w | 27.3±4.27 | 27.7±4.53 | 28.7±3.21 | 28.8±2.90 | 1,2,3,4,5,7,8,9,10,13 |
| Maryam et al.2012 | Iran | 30 | 31 | Omega-3 4×1g/qd | placebo 4×1g/qd | 8w | 27.33±4.27 | 27.73±4.53 | 28.67±3.21 | 28.77±2.92 | 1,2,3,4,5 |
| Ishwarpreet et al.2022 | India | 48 | 49 | Probiotic 10billion CFU/qd(2 months),bid(4 months) | placebo /qd(2 months),bid(4 months) | 24w | 23.6±3.9 | 24.4±4.8 | 26.5±5.3 | 27.8±4.8 | 1,2,3,4,5,11 |
| Z. E. et al.2018 | Iran | 21 | 21 | Probiotic/qw | placebo/qw | 8w | 29.52±5.82 | 30.60±7.43 | 26.29±1.70 | 26.77±1.70 | 1,2,3,4,5,6,11 |
| Arman et al.2022 | Iran | 45 | 43 | Probiotic 2×500mg/qd | placebo 2×500mg/qd | 12w | 28.13±5.48 | 28.95±5.13 | 32.88±6.11 | 31.99±4.22 | 11,12 |
| Z. E. et al.2019 | Iran | 22 | 21 | Probiotic/qd | placebo/qd | 8w | 29.52±5.82 | 30.60±7.43 | 26.29±1.70 | 26.77±1.70 | 7,8,9,10,13 |
| Elham et al.2020 | Iran | 50 | 49 | Probiotic 1000mg/qd | placebo 1000mg/qd | 12w | 28.1±5.5 | 29±5.1 | 32.89±6.11 | 32±4.23 | 1,2,7,8,9,10 |
| Elham et al.2018 | Iran | 50 | 49 | Probiotic 1000mg/qd | placebo 1000mg/qd | 12w | 28.1±5.5 | 29±5.1 | 32.89±6.11 | 32±4.23 | 3,4,5,6,13 |
| Tanaz et al.2015 | Iran | 32 | 33 | Probiotic 500mg/qd | placebo/qd | 8w | 26.5±0.1 | 25.72±0.1 | 26.06±0.1 | 25.8±0.1 | 3,4,5,6,13 |
| Shahnaz et al.2017 | Iran | 30 | 30 | Probiotic/qd | placebo/qd | 12w | 25.2±5.4 | 24.8±5.1 | 25.3±4.2 | 26.4±4.3 | 1,2,3,4,5,6,7,8,9,10, |
| Maryam et al.2018 | Iran | 30 | 30 | Probiotic/qd | placebo/qd | 12w | 27.2±4.6 | 27.7±4.7 | 23.7±3.6 | 23.6±3.5 | 1,2,11,12,13 |
| Batool et al.2019 | Iran | 34 | 32 | Selenium 200 μg/qd | placebo/qd | 12w | 29.4±5.3 | 28.6±5.5 | 28.3±5.2 | 29.5±5.4 | 1,2,7,8,9,10,11,12 |
| Zahra et al.2019 | Iran | 18 | 18 | Selenium 200μg/qd | placebo/qd | 8w | 32.1±4.7 | 32.6±3.5 | 27.2±3.1 | 28.6±2.5 | 1,2 |
| Fatemeh et al.2016 | Iran | 26 | 27 | Selenium 200μg/qd | placebo/qd | 12w | 29.23±0.96 | 28.90±1.17 | 29.4±0.88 | 28.39±0.72 | 3,4,5,11,12 |
| Mehri et al.2015 | Iran | 35 | 35 | Selenium 200μg/qd | placebo/qd | 8w | 25.4±5.1 | 25.7±4.8 | 25.0±3.7 | 25.2±4.1 | 1,2,3,4,5,6,7,8,9,10 |
| Shahrzad et al.2022 | Iran | 20 | 20 | Selenium 200μg/qd | placebo/qd | 8w | 32.6±4.6 | 32.8±4.1 | 25.2±4.1 | 25.6±2.5 | 1,2,3,4,5,6,7,8,9,10 |
| Shahrzad et al.2017 | Iran | 20 | 20 | Selenium 200μg/qd | placebo/qd | 8w | 31.1±4.7 | 31.4±3.6 | 26.5±4.1 | 27.3±2.6 | 1,2 |
| Shokoufeh et al.2012 | Iran | 15 | 16 | Vitamin D 0.5mg/qd | placebo/qd | 12w | 24.7±3.3 | 25.2±7.9 | 24.8±5.3 | 25.3±5.1 | 1,3,4,5 |
| Nazia et al.2014 | USA | 13 | 15 | Vitamin D 12,000IU/qd | placebo/qd | 12w | 28.2±5.2 | 28.7±5.6 | 37.20±4.53 | 35.09±9.81 | 2,3,4,5,6,7,8,9,10,11,13 |
| Christian et al.2018 | Austria | 81 | 42 | Vitamin D 20,000IU/qw | placebo/qw | 24w | 25.4±4.6 | 27.2±5.5 | 27.3±7.4 | 28.3±7.8 | 3,5,6,7,8,11 |
| Zeeshan et al.2019 | UK | 18 | 19 | Vitamin D 3200IU/qd | placebo/qd | 12w | 28.6±5.5 | 29.1±7.5 | 35.4±10.6 | 33.8±7.2 | 1,2,9 |
| H. Rahimi et al. 2013 | Iran | 24 | 26 | Vitamin D 50,000IU/every 20days | placebo/every 20days | 8w | 26.8±4.7 | 27±3.7 | 29.1±4.62 | 28.28±3.51 | 1,2,7,8,9,10,13 |
| Hania et al.2012 | Iran | 24 | 26 | Vitamin D 50,000IU/q20d | placebo/q20d | 8w | 26.8±4.7 | 27.0±3.7 | 29.1±4.62 | 28.28±3.51 | 1,2,3,4,5,6 |
| Majid et al.2018 | Iran | 20 | 20 | Vitamin D 50,000IU/qow | placebo/qow | 8w | 29.9±4.4 | 30.1±3.4 | 27.7±3.9 | 28.4±2.6 | 1,2,3,4,5,6,7,8,9,10 |
| Maryam et al.2017 | Iran | 35 | 35 | Vitamin D 50,000IU/qow | placebo/qow | 12w | 18-40 | 18-40 | - | - | 3,4,5,6,7,8,9,10,11,12,13 |
| Nahla et al.2020 | Jordan | 29 | 29 | Vitamin D 50,000IU/qw | placebo/qw | 12w | 23.6±4.3 | 23.9±6.0 | 27.3±1.9 | 26.9±1.6 | 3,4,5,11,12,13 |
| Mohamad et al.2015 | USA | 35 | 18 | Vitamin D 50,000IU/qw | placebo/qw | 8w | 30.5±1 | 29.6±1.7 | 30±1 | 28±1.6 | 5,7,8,9,10,11,12 |
| S. Jafari et al.2017 | Iran | 26 | 28 | Vitamin D 50,000IU/qw | placebo/qw | 12w | 28.43±6.27 | 27.83±5.71 | 31.13±4.99 | 31.61±4.91 | 1,2,11,12 |
| Maryam et al.2017 | Iran | 19 | 17 | Vitamin D 50,000IU/qw | placebo/qw | 8w | 26.21±4.62 | 22.76±4.40 | - | - | 3,4,5,6 |
| Outcome indicators:1.weight,2.BMI,3.FBG,4.FINS,5.HOMA-IR,6.QUICKI,7.TG,8.TC,9.LDL-C,10.HDL-C,11.TT,12.SHBG,13.CRP | | | | | | | | | | | |

**TableS3 The** **report of adverse reactions**

| **Study** | **Intervention** | **Adverse reactions** | |
| --- | --- | --- | --- |
|  |  | **T** | **C** |
| Arman et al.2022 | Probiotic | 8/45(8 people have flatulence) | 4/43 (4 people have flatulence) |
| Shokoufeh et al.2012 | Vitamin D | 2/15 (1 person has a headache and 1 person is non tolerance of drug) | 0/16 |
