# Supplementary material for: Comparison of nutritional supplements in improving glycolipid metabolism and endocrine function in polycystic ovary syndrome: a systematic review and network meta-analysis
Source: PeerJ. 2023 Nov 13;11:e16410. doi: 10.7717/peerj.16410 (PMC10652859; doi:10.7717/peerj.16410)
Supplement: Supplemental Information 3 [file peerj-11-16410-s003.docx]

**Systematic Review and/or Meta-Analysis Rationale**

1.The rationale for conducting the systematic review / meta-analysis.

In the clinical treatment of patients with PCOS, we have observed that many patients take nutritional supplements such as vitamin D, coenzyme Q10 and probiotics. After reading relevant clinical research literature, we became interested in the therapeutic effects of nutritional supplements on PCOS. We considered that direct comparisons of different nutritional supplements in managing PCOS remain inadequate, and the evidence related to the role of a broader range of nutritional supplements in PCOS need to be updated and evaluated.

2. The contribution that it makes to knowledge in light of previously published related reports, including other meta-analyses and systematic reviews.

The meta-analyses and systematic reviews published by Zhang, Zhao, Gong, Fazelian et al. evaluated the effects of nutritional supplements such as coenzyme Q10, vitamin E, inositol, vitamin D, selenium, carnitine, and chromium on the endocrine and metabolic status of PCOS. By summarizing the nutritional supplements that have been evaluated for effectiveness in these related reports, we selected 9 nutritional supplements included in this article for a network comparison. Meanwhile, the results in these literature are highly similar to some of the conclusions in our article, which to some extent also provides support for the credibility of this study’s conclusions.
